# Comparative analysis of ABCB1 reveals novel structural and functional conservation between monocots and dicots

**DOI:** 10.3389/fpls.2014.00657

**Published:** 2014-11-26

**Authors:** Amandeep K. Dhaliwal, Amita Mohan, Kulvinder S. Gill

**Affiliations:** Department of Crop and Soil Sciences, Washington State UniversityPullman, WA, USA

**Keywords:** ABCB1, orthologs, comparative analysis, gene structure, intron evolution, ligand binding, NBDs

## Abstract

Phytohormone auxin plays a critical role in modulating plant architecture by creating a gradient regulated via its transporters such as ATP-binding cassette (ABC) B1. Except for Arabidopsis and maize, where it was shown to interrupt auxin transport, ABCB1's presence, structure and function in crop species is not known. Here we describe the structural and putative functional organization of ABCB1 among monocots relative to that of dicots. Identified from various plant species following specific and stringent criteria, *ZmABCB1*'s “true” orthologs sequence identity ranged from 56–90% at the DNA and 75–91% at the predicted amino acid (aa) level. Relative to *ZmABCB1*, the size of genomic copies ranged from −27 to +1.5% and aa from −7.7 to +0.6%. With the average gene size being similar (5.8 kb in monocots and 5.7 kb in dicots), dicots have about triple the number of introns with an average size of 194 bp (total 1743 bp) compared to 556 bp (total 1667 bp) in monocots. The intron-exon junctions across species were however conserved. N-termini of the predicted proteins were highly variable: in monocots due to mismatches and small deletions of 1–13 aa compared to large, species-specific deletions of up to 77 aa in dicots. The species-, family- and group- specific conserved motifs were identified in the N-terminus and linker region of protein, possibly responsible for the specific functions. The near-identical conserved motifs of Nucleotide Binding Domains (NBDs) in two halves of the protein showed subtle aa changes possibly favoring ATP binding to the N-terminus. Predicted 3-D protein structures showed remarkable similarity with each other and for the residues involved in auxin binding.

## Introduction

Membrane transport proteins are crucial to facilitate transport of a wide spectrum of substrates across the lipophilic membrane. Passive transporters require a concentration gradient to transport solutes whereas active transporters require energy to move substrates across the membrane against a concentration gradient. Protein motifs, common to active transporters, are required for the binding of ATP as an energy source for transport of a wide range of substrates. First reported in bacteria to transport nutrients across the membrane (Berger and Heppel, 1974), ABC containing active transporters is the largest and perhaps one of the most diverse protein families. ABC family is characterized by the presence of a unique ABC signature motif in the NBD that is involved in substrate translocation (Hewitt and Lehner, 2003). The number of this motif varies among different proteins depending upon the fusion of NBDs (reviewed in Verrier et al., 2008). The individual NBD present in separate polypeptides particularly in prokaryotes and eukaryotic mitochondrial and endoplasmic reticulum is functional upon dimerization of two proteins (heterodimer). In eukaryotes, the two NBDs are fused in a single polypeptide (homodimer) to form a functional protein (Higgins, 1992).

The ABC family is further divided into eight subfamilies (A–H) based on the phylogenetic analysis of amino acid sequences of the NBDs (reviewed in Verrier et al., 2008). In plants, members of the subfamily B and C function mainly as exporters (Noh et al., 2001; Multani et al., 2003; Shitan et al., 2003; Santelia et al., 2005; Shi et al., 2007), D as importers (Subramani, 1998), and E in the suppression of RNA silencing (Sarmiento et al., 2006). The function of A and F family members has not yet been determined (reviewed in Verrier et al., 2008). No plant orthologs so far have been found for any of the H subfamily members.

The B subfamily is well studied in a wide range of organisms from bacteria to human (reviewed in Holland and Blight, 1999). One of its members, ABCB1, in humans provides resistance against anti-cancerous drugs thus is also referred as Multi Drug Resistance (MDR) protein (Willingham et al., 1986). The ABCB1 is expressed throughout the human body and plays a critical role in protecting the vital organs such as brain from toxic drugs/substrates (reviewed in Borst and Schinkel, 2013). Hence, ABCB1-substrate interactions are widely used for pharmacological studies. ABCB1 protein is known to have a similar function in mouse and *Caenorhabditis elegans* (Aller et al., 2009; Jin et al., 2012). The available crystalline structures of ABCB1 in mouse and *C. elegans* thus provide a platform to perform homology based modeling of the protein in other species (Ravna et al., 2009; Bailly et al., 2011).

In plants, the functional orthologs of human *ABCB1* (*HsABCB1*) were identified while searching for genes conferring cross resistance to herbicides (Dudler and Hertig, 1992). Later, studies in Arabidopsis showed ABCB1 protein to be involved in the transport of a growth regulating molecule—indole acetic acid (IAA, an auxin) (Noh et al., 2001; Geisler et al., 2005). Auxin plays a pivotal role in a variety of plant growth and development processes by creating a concentration gradient that is maintained via transporters (reviewed in Petrásek and Friml, 2009). There are more than 120 known putative ABC members in plants of which only few have been characterized (Sánchez-Fernández et al., 2001). ABCB-1, −4, −14, −15, and −19 have been involved in Polar Auxin Transport (PAT) as their mutants showed reduced auxin transport (Noh et al., 2001; Kaneda et al., 2011; Kubeš et al., 2012). The other members were identified by homology search without any functional characterization. However, a detailed structural and functional analysis of these transporters in crop plants is lacking.

Plant height is an important agronomic trait controlled by various growth hormones. The height reduction in cereals has been achieved mainly by manipulating gibberellins (GA) (Peng et al., 1999); and to some extent by brassinosteroids (BR) (Saisho et al., 2004). The GA-mutants have been extensively utilized to reduce plant height in wheat, maize and rice causing genetic uniformity. Moreover, mutants in GA and BR have been associated with negative effects on various agronomic traits (Fick and Qualset, 1976; Keyes et al., 1990; Saisho et al., 2004). Alternatively, plant height can also be regulated by modulating auxin transport as was reported in maize, sorghum and Arabidopsis (Noh et al., 2001; Multani et al., 2003; Geisler et al., 2005). The mutation in maize (*ZmABCB1*) and sorghum (*SbABCB1)* reduced plant height by compacting the lower internodes without any adverse effect on rest of the plant (Multani et al., 2003). A mutation in the Arabidopsis ortholog of this gene showed no effect on plant height under long day conditions but exhibited intermediate dwarf phenotype under short day conditions (Noh et al., 2001; Geisler et al., 2005). These differences in the gene effect could be due to the differences in plant architecture and anatomy between monocots and dicots. The detailed structure, function and evolution of ABCB1; and its role in controlling plant height or other important agronomic traits, is however not well known in crop species.

Objective of the present study was to identify and understand structure, putative function and evolution of true orthologs of *ZmABCB1* from various plant species by a detailed bioinformatics analyses.

## Materials and methods

### Identification of true orthologs

Because the *ABCB1* gene was functionally characterized in maize, predicted protein sequence of *ZmABCB1* was used as a reference to identify the true orthologs in different species. The species were considered on the basis of their agronomic importance, sequence information availability, plant anatomy (monocots vs. dicots), and diverse phenotypes. For a sequence to be considered as a true ortholog, we developed the following criterion that should be met: (1) The highest level of sequence identity and query coverage along the protein length; (2) Presence of all domains and motifs present in the original query sequence; (3) The retrieval of ZmABCB1 sequence at first place when obtained orthologous sequence is used as a query; and (4) The similarity in relative size and distance among various motifs and domains to that of the query sequence. The cDNA and full length gene sequences of true orthologs were retrieved for *Sorghum bicolor* (sorghum, AY372819.1); *Hordeum vulgare* (barley, BAJ99824.1); *Oryza sativa* (rice, BAD12940); *Brachypodium distachyon* (Brachypodium, XM_003571225); *Arabidopsis thaliana* (Arabidopsis, NM_129247.2); and *Glycine max* (soybean, XM_003554341) from NCBI (http://www.ncbi.nlm.nih.gov) and Gramene (http://www.gramene.org/) databases (as on September 2012). To generate the full length *ABCB1* sequence from wheat (*Triticum aestivum*), wheat Expressed Sequence Tags (ESTs) identified as orthologs of ZmABCB1 sequence were used as queries against “draft assembly of gene rich regions” of Chinese spring (http://www.cerealsdb.uk.net/cerealgenomics/CerealsDB/search_reads.php). The obtained contigs were assembled using cap3 to synthesize a consensus sequence, which was again used as a query to search the database for a maximum length of contig. This procedure was repeated until overlapping contigs were obtained. The retrieved overlapping contigs were assembled into a full length *TaABCB1* genomic copy. After marking intron-exon boundaries, the resultant cDNA sequence was translated into protein sequence (http://web.expasy.org/translate/). Similarly, true orthologs of Arabidopsis ABCB19 (AEE77498.1) were identified from NCBI in maize (NP_001169660.1), sorghum (XP_002447959.1), barley (BAK05967.1), wheat (AK332498), Brachypodium (XP_003579896.1), rice (LOC_Os04g38570.1), and soybean (XP_003543769.1). The sequences for AtABCB4 (O80725.1), AtABCB14 (AEE30902.1) and AtABCB15 (Q9LHD1.1) were also retrieved from NCBI for phylogenetic analysis.

### Gene structure analyses

The exon/intron junctions and translation start and stop sites of *ABCB1* gene in all studied species were predicted by aligning the available cDNA sequences with their respective genomic sequences using multiple sequence alignment tool-ClustalW2 (http://www.ebi.ac.uk/Tools/msa/clustalw2/) with default settings. In case of wheat, the exon/intron junctions and translation start and stop sites were marked by aligning the assembled *ABCB1* genomic sequence with cDNA of closest relative specie barley. The maize exons were aligned with exon sequences of other species using “Align X” module of the *Vector NTI Advance*™ 11.0. On the basis of sequence homology with maize, the exons in different species were highlighted with different colors to study the intron-exon structure of gene. The location of intron insertion within a codon was marked as intron phase zero, one and two. The intron phase distribution of zero, one and two is defined as the location of intron between two codons, after the first nucleotide and second nucleotide of a codon of exon respectively.

### Analyses of predicted amino acid sequences of ABCB1 orthologs

To analyze the differences and similarities among monocots and dicots, predicted aa sequences of all the eight species were aligned using “Align X” module of the Vector NTI to generate a consensus sequence. The aa with maximum occurrence across species at a position was considered in consensus sequence. The positions where all aa were different among species, maize aa were considered at those positions to generate a consensus sequence. The sequence insertions present in one or more species were also included in the consensus sequence. Monocots and dicots were aligned separately with the generated consensus sequence. For each position, a scale of 1–6 for monocots and 0–2 for dicots was used to represent aa similarity with the consensus sequence. The value of six in monocots and two in dicots indicates the presence of similar aa at a particular position, whereas zero refers to no similarity with respect to consensus sequence. The deletions present in species corresponding to consensus sequence were marked above the similarity plot. Exons on the consensus sequence were marked respective to maize sequence. The CDD analysis identified domains and motifs in consensus protein sequence (http://www.ncbi.nlm.nih.gov/Structure/cdd/wrpsb.cgi).

### Phylogenetic analyses

The predicted protein sequences of all species were used to perform phylogenetic analysis with MEGA 5.05 software (Tamura et al., 2011). The evolutionary relationship was inferred by the Neighbor-Joining method of distant matrix and the unrooted phylogenetic tree was built using a bootstrap analysis of 1000 replicates.

### 3-dimensional protein analyses

Predicted ABCB1 protein sequences of all species were used to generate the 3-D structure models using CePGP1 as a reference model on PHYRE2—Protein HomologY Recognition Engine [www.sbg.bio.ic.ac.uk/phyre2/; (Wass et al., 2010)] using Normal mode for modeling with default settings. The search engine is based on SCOP and PDB, and HMM-HMM alignment techniques for remote homology detection. The protein model generated by the PHYRE2 was used to predict ligand-binding site by the 3DLigandSite (http://www.sbg.bio.ic.ac.uk/3dligandsite) (Wass et al., 2010). The 3DLigandSite predicted binding sites based on the comparison of query structure with a binding site library, which is extracted from protein–ligand complexes, to select similar structures followed by superimposition of the ligands onto the query structure to identify the binding sites. The ligand binding analysis identified three–five clusters in all the species. The cluster having maximum number of ligands was selected for the prediction of ligand binding in target sequence.

## Results

### Retrieving true orthologs of *ZmABCB1*

Since the ABCB1 protein was originally characterized in *C. elegans* and mouse and its gene function from plants was known in maize (Multani et al., 2003), the longest possible reading frame of predicted ZmABCB1 protein sequence was used as a query to identify its true orthologs from other plant species, following the approach and criteria described in the “materials and methods” section. The orthologous sequences from Arabidopsis and sorghum were available at NCBI (Dudler and Hertig, 1992; Multani et al., 2003). Orthologous sequences from other plant species were identified by tBLASTn (search translated nucleotide database using protein query) comparison with default parameters including *E*-value of 10 against the non-redundant nucleotide database of NCBI.

Multiple sequences were identified each for barley, rice, Brachypodium, and soybean exhibiting a wide range of identity and coverage. A true ortholog was identified for each of the species following the criteria outlined in the “materials and methods” section. The true orthologs often showed an *E*-value of 0 along with the highest level of coverage and identity throughout the protein length. Since the full length *ABCB1* ortholog was not available in the wheat database, a complete orthologous genomic copy was assembled by combining various available genomic sequences including Chinese Spring (CS) 5X genomic database (Wilkinson et al., 2012) (see Materials and Methods). Initially, the tBLASTn comparison identified 100 wheat ESTs of which CJ800530 and CA730599 were identified to be orthologs of ZmABCB1, respectively showing 72 and 79% aa sequence identity. The BLASTn (search nucleotide database using nucleotide query) search for “draft assembly of gene rich regions” of the 5X wheat database has identified one contig for CA730599 and five for CJ800530. Further sequence walking with these contigs identified two large contigs that eventually assembled into a full length *TaABCB1* genomic copy. The intron-exon boundaries in *TaABCB1* were marked according to barley (*HvABCB1*) genomic and cDNA sequence, the closest species to wheat.

### Structure of *ABCB1* gene

**Figure 1:**
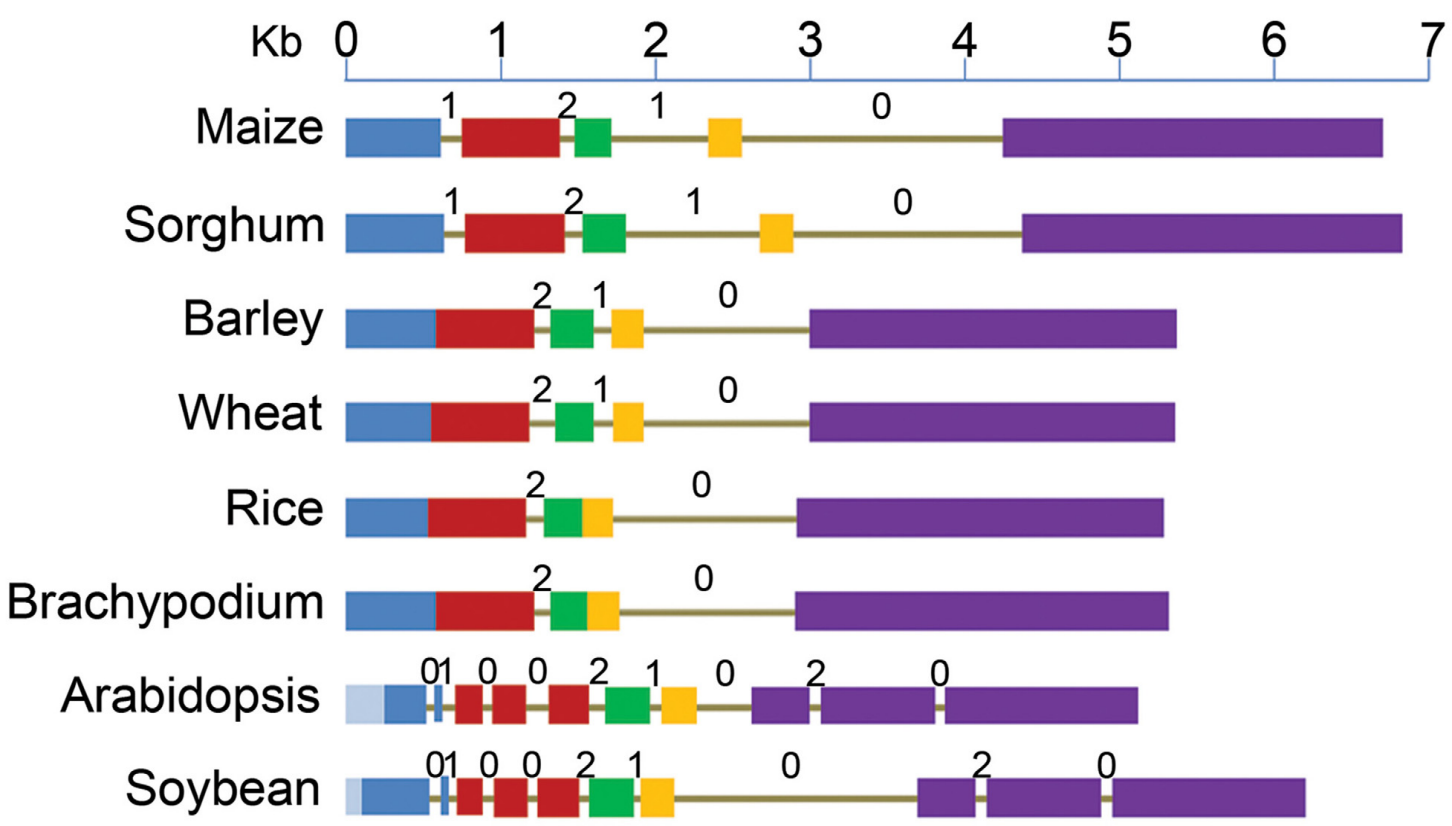
**Structure of *ABCB1* genes starting from translation start to stop sites in six monocot and two dicot species**. Drawn to scale, exons are represented by boxes and introns as lines. Each of the five exons of maize is shown by different colors and the corresponding sequence in other species is marked by the same color. Deletions (light blue) in exon 1 of dicots are marked toward 5' end. Intron phases 0, 1, and 2 are marked above each intron.

The genomic orthologs from studied species were analyzed for their structure in comparison to the *ZmABCB1*. The genomic sequence of sorghum was the largest and that of Arabidopsis was the smallest (Figure 1). In monocots, the smallest genomic copy was present in rice and in dicots, the largest copy was present in soybean. The gene length variation between monocots and dicots was mainly due to the differences in size and number of introns and deletions in exon 1 (Figure 1). An average intron size of 194 bp in dicots was 2.8 times smaller than the average intron size of 556 bp in monocots. Neither the total intronic sequence length (1743 bp) nor the size range (1–2.5 kb) among the dicot species was much different from that of the monocots with the corresponding average length of 1667 bp and the range of 1.3–2.6 kb. The size of exonic regions between the two groups was similar although the overall regions were slightly smaller in dicots. The size of exonic region in monocots ranged from 4.0 kb to 4.2 kb compared to 3.9 kb to 4.0 kb in dicots.

Major differences were observed among various plant species for the number and location of introns (Figure 1, Table S1). Number of introns in dicots was nine compared to two–four in monocots. The reference gene, *ZmABCB1*, and that from sorghum have four introns each with one at the 3' end being the largest. Wheat and barley has three introns compared to two each in rice and Brachypodium.

Among monocots, maize and sorghum genes showed the largest exomes, while that from rice and Brachypodium showed the smallest. Exon 1 in dicots showed the largest variation in size due to deletions of 284 bp in Arabidopsis and 108 bp in soybean (Figure 1). Relative to maize, additional introns were present in exons 1, 2, and 5 of the dicots (Figure 1). On the other hand, maize exon 1 and 2 were fused in all other monocots except in sorghum. Similarly, the exon 3 and 4 were fused in rice and Brachypodium (Figure 1). However, similar adjacent exonic sequences were present at positions in other monocots where introns were absent corresponding to maize and sorghum. The common intron-exon boundaries remained highly conserved in all studied species.

### Intron evolution

Intron phase is the position of intron within a codon of an exon. Introns can insert in a sequence between two consecutive codons (phase zero intron), between first and second nucleotide of a codon (phase one intron) and between second and third nucleotide of a codon of an exon (phase two intron) (Figure 1). The introns common among species have the same phase distribution. The maize introns 2 and 4, present in all studied species have phase distribution of 2 and 0 respectively (Figure 1). Maize intron 1 was absent in the rest of the monocots except sorghum, as well as intron 3 that was absent in rice and Brachypodium, has a phase distribution of 1. Lineage specific introns (1, 3, 4, 8, 9) in Arabidopsis and soybean have phase 0 distribution except intron 8 that has a phase distribution of 2.

The *ZmABCB1* intron 4 (1663 bp) showed sequence identity ranging from 44.7% in Arabidopsis to 86.8% in sorghum. It was the largest intron present in the studied species maintaining a similar size except in Arabidopsis where the intron was about five times smaller due to large deletions (Table S1). The most uniformly sized *ZmABCB1* intron 2 present in all of the species showed a sequence identity ranging from 31.9% in Arabidopsis to 51.8% in sorghum. Intron 3 showed the maximum variation in size ranging from 1.3 to 9.7-fold among various species (Table S1). The intron loss in monocots occurred from 5' and middle of the gene whereas intron insertion in dicots happened mainly at the 3' and 5' ends (Figure 1).

### DNA sequence comparisons

The *ZmABCB1* genomic copy showed 90% sequence identity to that of sorghum, 80% to both wheat and barley, 78% to both rice and Brachypodium, 62% to Arabidopsis, and 56% to soybean (Table S2). Similar comparison of the *ZmABCB1* cDNA with sequences of other species showed an identity of 93% to sorghum, 86% to rice, 85% to both wheat and barley, 82% to Brachypodium, 67% to Arabidopsis, and 66% to soybean. The exonic region in monocots corresponding to maize exon 1 showed the lowest sequence similarity ranging from 67.5% in rice to 84% in sorghum; whereas, in Arabidopsis and soybean, the first two exons corresponding to the maize exon 1 have 64 and 61% sequence identity, respectively (Table S2). Maize exon 2 has the maximum sequence similarity to the corresponding regions in the monocots with a range of 84.2–94.5% compared to 65–68% identity in the dicots.

### Analyses of predicted ABCB1 ortholog amino acid sequences

**Figure 2:**
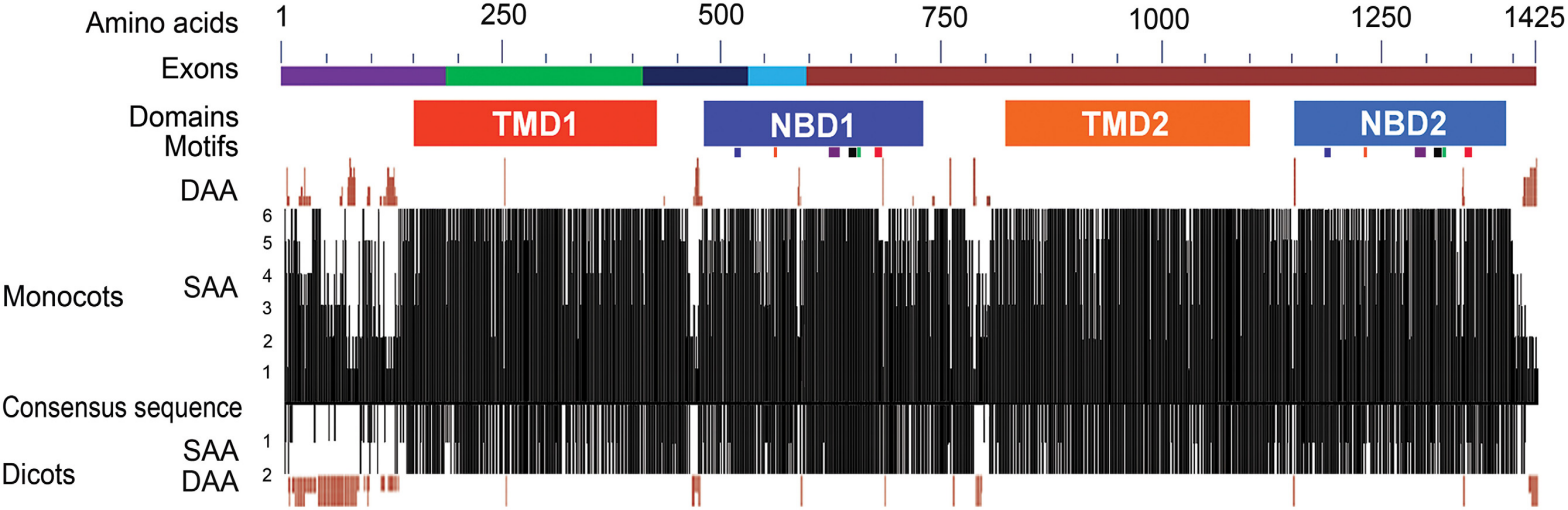
**Structural features and sequence similarity of the predicted ABCB1 proteins**. The sequence differences in NBDs and TMDs between two halves of ABCB1 proteins are depicted with shades of different colors. The exons marked with different colors above the consensus sequence are drawn according to that of maize. The six known motifs (from left to right in each NBD—Walker A, Q-loop, ABC transport signature, Walker B, D-loop, and H-loop motifs) are depicted as colored boxes under each of NBD1 and NBD2. The presence of similar amino acids against consensus sequence was plotted on a scale of 1–6 in monocots and 0–2 in dicots. The deletions of aa in different species compared to consensus sequence were marked on either side of the similarity plot. DAA and SAA refer to deleted and similar amino acids.

The predicted protein size of the *ABCB1* ranged from 1286 aa in Arabidopsis to 1402 aa in sorghum. Overall, the predicted ZmABCB1 protein sequence showed aa sequence identity of 91% with sorghum, 85% with rice, 83% with wheat and barley, 79% with Brachypodium, 76% with soybean and, 75% with Arabidopsis (Figure S1 and Table S3). To identify the monocot-dicot group specific differences, six monocot and two dicot species were individually aligned with a consensus sequence of the ABCB1 protein (see Materials and Methods). Maximum aa mismatches and deletions occurred at the N- and C-termini, “linker” region present between the two halves of the protein and the region between Trans membrane Domain (TMD) and NBD in each half of the protein (Figure 2 and Figure S1). In the monocots, differences at the N-terminus were mainly due to aa mismatches and deletions of up to 13 aa in rice. These differences were less pronounced between maize and sorghum, and between wheat and barley. The predicted aa of rice showed relatively more sequence similarity with maize, sorghum, and Brachypodium than with wheat and barley. The N-terminus of the monocots contained proline rich motifs of two–six residues. The aa differences were less pronounced at C-terminus between maize and sorghum, wheat and barley, and Arabidopsis and soybean. Compared to monocots, the N-terminus of dicots has more aa deletions, for example, a 77 aa long deletion was present in Arabidopsis (Figure 2 and Figure S1).

The Conserved Domain Database (CDD) analysis of the ABCB1 consensus sequence (see Materials and Methods for consensus sequence) was performed to annotate the functional motifs and domains of the protein. ABCB1 protein is a bipartite structure each having TMD and NBD (Figure 2). These domains were organized as TMD1-NBD1-Linker-TMD2-NBD2. The two halves of the protein were connected with a linker region of about 101 aa present between aa 723 and 823 (Figure S1). We identified previously unknown motifs of variable lengths with a maximum of 15 aa in the linker region that were conserved among all of the studied species. In addition to sequence changes among species in the variable region of the linker, Brachypodium carried insertion of maximum four aa and rice showed deletion of five aa. Overall, protein sequences were relatively more similar between maize-sorghum, wheat-barley and Arabidopsis-soybean.

The region present between TMD1 and NBD1 (431–479) has similar aa sequence among all species except for few insertions and deletions of about 6 aa (Figure S1). Similarly, along with aa sequence similarity among the species in the region between TMD2 and NBD2 (1100–1148), insertion of 2 aa was present in Brachypodium. The sequences present between TMD1-NBD1 and TMD2-NBD2 have no aa similarity with each other (Figure S1).

**Figure 3:**
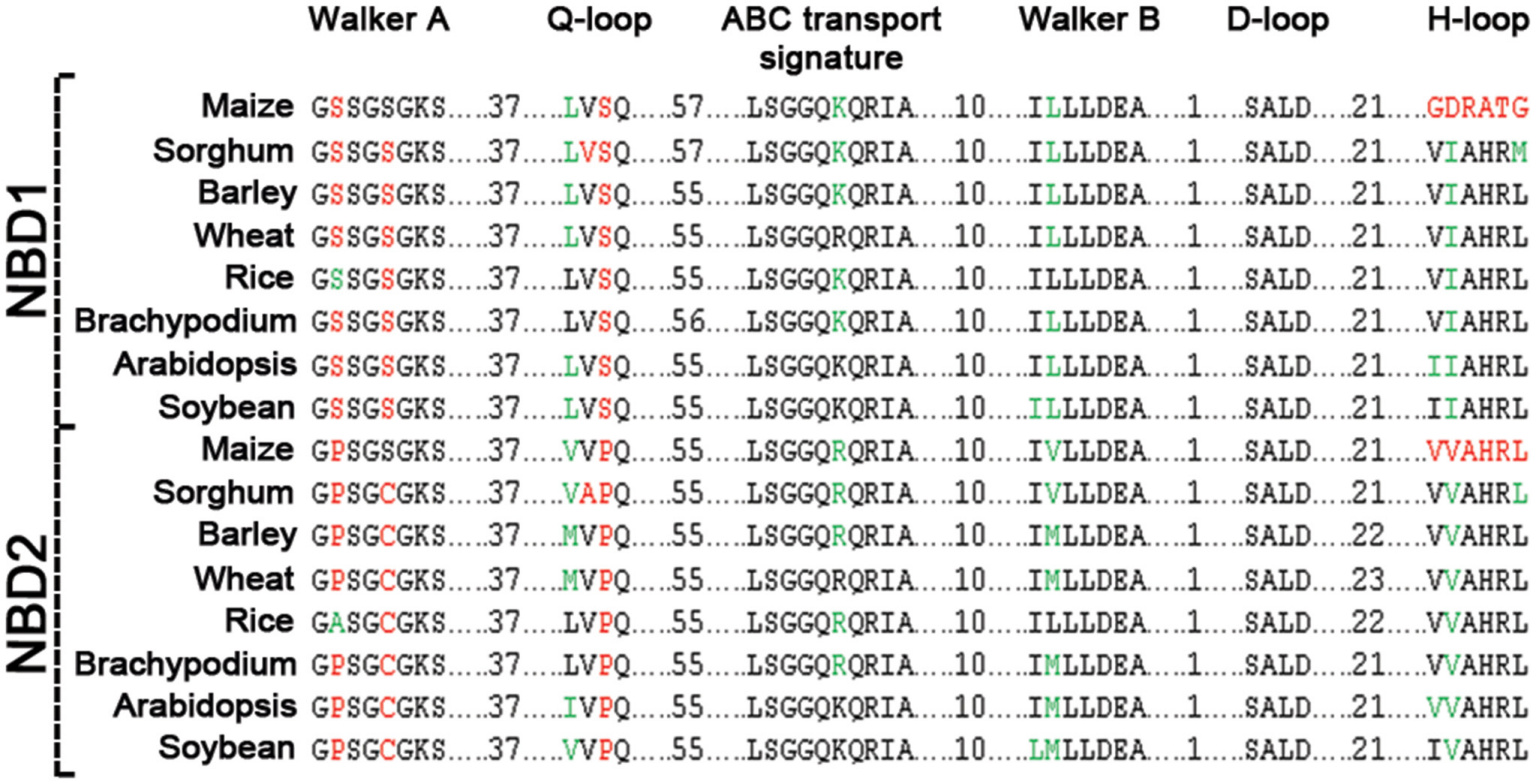
**Sequence comparison of motifs between NBD1 and NBD2 of ABCB1**. Conserved residues between two domains are highlighted in black, synonymous changes in green and non-synonymous changes are in red.

Comparison of the two NBDs in ABCB1 showed aa identity of 60.4% in soybean, 59.3% in rice, 58.5% in Brachypodium, 56.6% in sorghum, 55.4% in barley and wheat, 55.1% in maize, and 48.1% in Arabidopsis (Table S3). Each NBD was consisted of six motifs including Walker A, Q-loop, ABC transport signature, Walker B, D-loop, and H-loop/switch (Figure 3). Interestingly, the number of aa between Walker A and Q-loop; ABC transport signature and Walker B; and Walker B and D loop for NBD1 (N terminal) and NBD2 (C terminal) were conserved among all species (Figure 3). The aa sequence identity for all the species between NBD1 and NBD2 was around 56%. However there was a small variation of two aa between Q-loop and ABC transport signature in NBD1 and two aa between D-loop and H-loop/switch motifs in NBD2 (Figure 3). Both synonymous (green) and non-synonymous (red) aa changes were observed between motifs of NBD1 and NBD2. Serine present at the second position of Walker A motif in NBD1 was substituted with non-synonymous proline in NBD2 except in rice, where it was substituted with synonymous alanine. Similarly, serine at the fifth position of Walker A was substituted with a non-synonymous cysteine except in maize. Serine at the third position in Q-loop of NBD1 has non-synonymous substitution with proline in NBD2 in all species. Except in maize, the H-loop was conserved among all species (Figure 3).

In monocots, the sequence of NBD1 was relatively less conserved (70.4%) than NBD2 (77.7%) whereas in dicots, the NBD1 (94.6%) was more conserved than NBD2 (86.3%) resulting in an overall aa identity of 67.9% in NBD1 and 63.6% in NBD2 among all species. Overall, the TMD1 showed aa sequence identity of 59.3% compared to 65.9% for TMD2 (Figure 2). The sequence identity of TMD1 and 2 in monocots was 74.3 and 73.6%, respectively compared to 70.6 and 92.4% in dicots. The sequence identity between TMD1 and TMD2 was 25% and between NBD1 and NBD2 was 56%.

Studied in AtABCB1, total 52 predicted aa were involved in auxin binding [as reported in (Bailly et al., 2011)], 17 of these were present between aa 58 and 323 in TMD1 and 35 were present between aa 710 and 980 in TMD2 of Arabidopsis (Table S5). These aa and their positions were conserved in all species except for two non-synonymous substitutions of leucine and phenylalanine at position corresponding to 713 and 716 of Arabidopsis in maize and sorghum.

### Phylogenetic analyses

**Figure 4:**
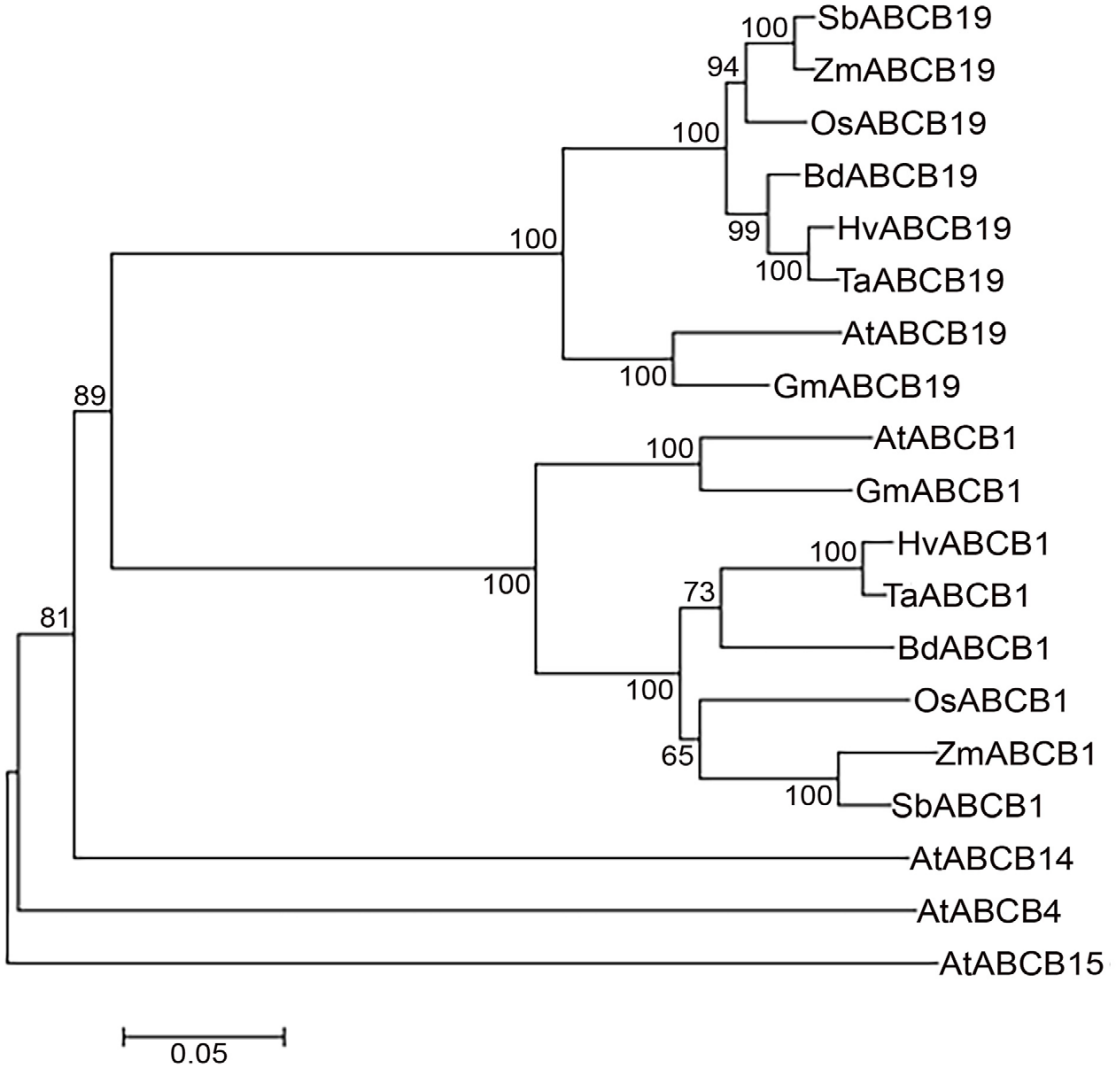
**Neighbor-Joining phylogenetic tree showing relationship between monocots and dicots ABCBs**. *Hordeum vulgare* (Hv), *Triticum aestivum* (Ta), *Brachypodium distachyon* (Bd), *Oryza sativa* (Os), *Zea mays* (Zm), *Sorghum bicolor* (Sb), *Arabidopsis thaliana* (At), and *Glycine max* (Gm). Scale bar represents the number of amino acid changes per site.

The phylogenetic analyses revealed distinct cluster of ABCB1 from ABCB-19, -4, -14, and -15 in plants (Figure 4). Within the ABCB1 clade, two distinct clusters corresponding to monocots and dicots were formed, with monocots further comprising of two sub-clusters. At a bootstrap value of >60, wheat, barley, and Brachypodium formed one sub-cluster whereas rice, maize and sorghum were grouped as a separate sub-cluster (Figure 4). The branch length marked the sequence changes in species relative to its common ancestor. Within sub-cluster 1, wheat-barley showed less divergence as indicated by a shorter branch length compared to Brachypodium (Figure 4). Similarly in sub-cluster 2, maize and sorghum showed less divergence compared to rice. However maize has accumulated more aa changes compared to sorghum as shown by its branch length (Figure 4).

### 3-dimensional protein analyses

**Figure 5:**
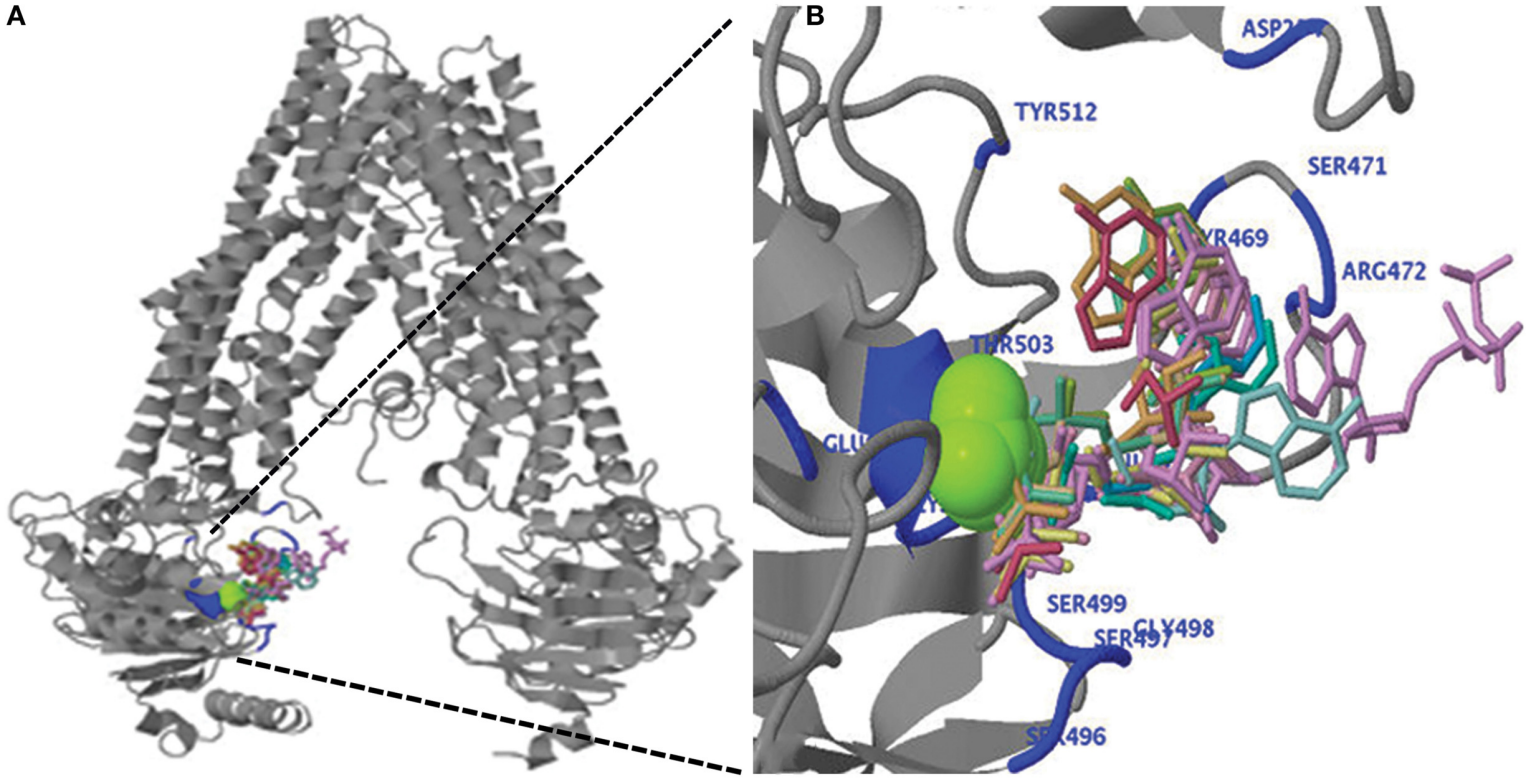
**(A)** Predicted three-dimensional structure of the ZmABCB1. **(B)** Ligand binding of magnesium ion (green), ADP and ATP (wired structure) as well as residues involved in binding (blue) are shown at the N-terminal region of ZmABCB1.

The *C. elegans* Phosphoglycoprotein1 (PGP1, also known as ABCB1) crystalline structure (PDB ID code: 4f4c) was utilized as a homology modeling template on “PHYRE2” to generate 3-D structures of various ABCB1 proteins. Using the CePGP1 as a single highest scoring template, 87–94% of ABCB1 query sequences from all species were modeled with 100% confidence. The ABCB1 query sequences showed 36% identity with the template sequence of CePGP1. In all studied species, the 3-D structure models revealed a V-shaped inward conformation consisting of two TMDs and two cytosolic NBDs similar to that in CePGP1 (Jin et al., 2012) (Figure 5A). The TMDs were composed of only α-helices whereas the NBDs were made up of both α-helices and β-sheets. The alignment of the secondary structure of CePGP1 with ABCB1 from plants showed similar pattern of α-helices and β-sheets. The structural features were conserved throughout the protein length except at the N-terminus and the linker region to which the corresponding regions were missing in *C. elegans* (data not shown).

We performed the ligand binding analysis (see Materials and Methods) to predict binding of various ligands to NBDs. Three–five predicted clusters were obtained for the binding of ATP, ADP, and magnesium (Mg) ligands. The cluster carrying the maximum number of ligands was selected for analysis. The ligands have shown preferential binding to the NBD present at the N terminal in all the species (Figure 5B). The aa involved in the ligand binding from TMD1 and NBD1 were highly conserved among all species (Table 1). Previously unknown motifs and residues involved in ligand binding were identified along with known Walker A and B motifs. A conserved residue from NBD1 (isoleucine) was present in all the species except with the synonymous substitution (valine) in Brachypodium. Of the identified novel motifs, one was present in TMD1—unknown motif (UM) 1 (FFD) and two were present in NBD1—UM2 (YPSRP) and UM3 (ERFY) (Table 1). Further in all the species, the position of UM1 and UM2 from Walker A was highly conserved whereas UM1 and UM2 were present at a variable distance of 234–240 aa and UM3 and Walker B at a distance of 111–113 aa (Table 1).

**Table 1:** **The predicted residues involved in ATP, ADP, and Mg binding in 3-D structures of ABCB1 in different species**.

**Species**	**TMD1**	**NBD1**													
	**UM1**	**UM2**			**Residue**	**Walker A**								**UM3**	**Walker B**
**Zm**	D	Y	S	R	I	S	S	G	S	G	K	S	T	Y	E
	234	469	471	472	477	496	497	498	499	500	501	502	503	512	626
**Sb**	D	Y	S	R	I	S	S	G	S	G	K	S	T	Y	E
	238	479	481	482	487	506	507	508	509	510	511	512	513	522	636
**Hv**	D	Y	S	R	I	S	S	G	S	G	K	S	T	Y	E
	224	462	464	465	470	489	490	491	492	493	494	495	496	505	617
**Ta**	D	Y	S	R	I	S	S	G	S	G	K	S	T	Y	E
	225	460	462	463	468	487	488	489	490	491	492	493	494	503	615
**Os**	D	Y	S	R	I	S	S	G	S	G	K	S	T	Y	E
	205	441	443	444	449	468	469	470	471	472	473	474	475	484	596
**Bd**	D	Y	S	R	V	S	S	G	S	G	K	S	T	Y	E
	223	461	463	464	469	488	489	490	491	492	493	494	495	504	617
**At**	D	Y	S	R	I	S	S	G	S	G	K	S	T	Y	E
	139	377	379	380	385	404	405	406	407	408	409	410	411	420	532
**Gm**	D	Y	S	R	I	S	S	G	S	G	K	S	T	Y	E
	189	427	429	430	435	454	455	456	457	458	459	460	461	470	582
**Ce**	D	Y	S	R	I	S	S	G	C	G	L	S	T	Y	E
	188	425	427	428	433	452	453	454	455	456	457	458	459	468	580

*Amino acids are represented as single letter abbreviations along with their positions in protein*.

## Discussion

ABC superfamily, characterized by the presence of ABC signature motif along with Walker A and B, H-, D- and Q-loop in NBD, is categorized into several subfamilies (named A through H) based on the variation in the NB domains (Higgins, 1992; reviewed in Verrier et al., 2008). Even though the NB motifs were conserved between prokaryotes and eukaryotes, the proteins have been implicated in diverse biological functions (Higgins et al., 1986). In ABCB1, the TM and NB domains account for about 74% of the total protein length. These domains are also present in many other proteins with functions unrelated to that of ABCB1 (reviewed in Verrier et al., 2008). Therefore, DNA or protein sequence comparisons will identify both orthologs as well as other related proteins. It is thus imperative to develop a precise protocol to identify true orthologs. Here we report such an approach using the *ABCB1* gene as an example.

The proposed criterion is particularly important for the gene family with a high level of sequence similarity among its members. For example, ABCB1 and ABCB19 proteins encoded similar domains and motifs and have shown high sequence coverage (Table S4). The true orthologs of ZmABCB1 from the eight species have shown high aa sequence coverage as well as identity (Table S3, S4). Further, the sequence evaluation of these two proteins identified a sub-region (linker) that can differentiate these two proteins. This linker region was highly dissimilar among species for these two proteins but was highly similar within ABCB1 and ABCB19 (Figure S2A). Similarly, irrespective of the highly conserved NB motifs among AtABCB-1, −4, −14, −15, and −19, the linker region was found to be highly variable among all these protein sequences (Figure S2B) thus linker region might play an important role in their specific functions.

Although the size of predicted ABCB1 protein was highly similar among various plant species, comparison of the genomic sequences revealed novel pattern of intron evolution. Interestingly, the total size of exome among various plant species was not much different but major differences were observed for the number and average size of the introns (Figure 1). Number of introns was similar among the dicot species but was different among monocot species (Figure 1). This might be due to rapid evolution of introns in monocots that resulted in intron loss whereas a similar number of introns were observed even in the evolutionary diverge dicot species including poplar (Carraro et al., 2012), *Vitis vinifera* and *Phaseolus vulgaris* (data not shown) (Roy and Gilbert, 2006). Irrespective of variable number of introns, the common intron positions were conserved between the two groups suggesting that all the introns in monocots were part of their ancestral genome (Trapp and Croteau, 2001). Although the range of total intron size remained same in monocots (ranged from 1.2 to 2.6 kb) and dicots (ranged from 1 to 2.5 kb), large differences for intron size were observed within groups. Changes in the number and average size of the introns without significantly affecting the protein sequence or structure suggest that at least for the *ABCB1* gene, evolution of introns is different from that of exonic regions.

Interestingly, the common intron-exon boundaries were highly conserved among the studied species suggesting that insertions or deletions are the main processes of intron evolution and not transposition to a new location within the same gene. Recently evolved monocots showed less number of introns compared to dicots suggesting intron deletions (Roy and Gilbert, 2005) but it is difficult to explain how the total size of the intronic region was maintained during such deletions. In the *ABCB1* gene, within monocots, after the divergence of Panicoideae from Poaceae, the first intron was lost in Pooideae and Ehrhartoideae and second intron was lost due to independent events in rice and Brachypodium (Roy and Gilbert, 2005). In *ABCB1*, introns were lost during the course of evolution as ancient common ancestor- *Amborella trichopoda* of angiosperms has nine introns and were maintained in old (soybean) to recently (Arabidopsis) evolved dicot species (Amborella, 2013).

For some genes it has been reported that introns insert preferentially at the protosplice site [(A/C) AG|G; (Rogozin et al., 2005)]. The intron insertion in the *ABCB1* gene did not show this preference for protosplice site as only 39% of the introns inserted at this site. The majority of the introns were present at the non-protosplice sites including 33% of the intron insertion at N|G, 19% into G|N, and 6% into N|N, where N is A, T and C nucleotide. Defined as intron insertion among three possible locations within a codon, phase distribution appeared to deviate significantly from a random distribution. About 44% of the insertions showed phase zero distribution, which is significantly higher than the expected 33%. The insertions at phase one and two were 28% each (Figure 1). Even the dicot-specific introns showed the same bias toward phase zero distribution. The major differences for the gene structure and protein sequences among species followed their evolutionary divergence. The major changes were observed among monocots and dicots (Figure 1 and Figure S1) that diverged about 200 million years ago (MYA) (Wolfe et al., 1989). Comparatively, less aa changes have been accumulated within recently diverged subgroups including wheat-barley (11.6 MYA), maize-sorghum (15–20 MYA) (Figure 4) (Chalupska et al., 2008). ABCB1 in Brachypodium has shown more sequence similarity with barley, corresponding to its recent divergence (32–39 MYA), than rice (40–53 MYA) and sorghum (45–60 MYA) (Initiative, 2010).

Even with the variation in gene structure, the domain and motif organization of ABCB1 remained conserved for each member of the gene family across various species; possibly retaining the primary function over the course of evolution. Sequence changes mainly occurred in the regions outside the functionally important parts of the protein (TMDs and NBDs) (Figure 2). Differential pattern of sequence similarity was observed throughout the protein length. The N-terminus was the most variable region of the protein and changes for both size as well as sequence were observed. The changes in the N-terminus occurred independently among groups, families and species. Despite sequence divergence, the N-terminus appeared to be functionally important as we have identified a novel monocot specific ten aa long motif (PELEAFHLPS, Figure S1). Additionally, a four aa long histidine rich motif known in substrate binding was identified at the N-terminus of the protein to be specific for rice (Weng et al., 2003). Arabidopsis showed a 77 aa long deletion at the N-terminus. The variable N-terminus was devoid of any signal peptide or pro-protein signatures. As suggested earlier (Williamson, 1994), this region may have a role either in binding or recognition of substrate as it contains proline residues. The C-terminus of the protein contains group, sub-group, and species-specific changes. Arabidopsis has a putative AMP-binding domain signature (IGMTSGSSSRVK) at the C-terminus. The 3-D structure in studied species attained the similar conformation as of ZmABCB1 with 98–100% structural similarity (ePDBFold; Krissinel and Henrick, 2004). Further, the auxin binding residues predicted through *in silico* analysis in AtABCB1 (Bailly et al., 2011) were also conserved in the identified orthologs suggesting their involvement in auxin transport.

NBDs have been proposed to bind ATP that is needed for an active transport (Davidson et al., 2008). Sequence comparison of the two NBDs in ABCB1 protein revealed that six motifs present in NBD1 were more conserved among plant species than NBD2 motifs (Figure 3). Detailed comparison identified subtle differences between the motifs of two domains possibly reflecting differences in their functions. Compared to NBD1, NB motifs including Walker A, Q-, and H-loops have sequence changes in NBD2. These motifs are believed to be involved in ATP binding and hydrolysis. All six motifs were highly conserved in NBD1 but have some aa changes in NBD2 (Figure 3). The distance among motifs was also more conserved in NBD1 except for the distance between Q-loop and ABC transport signature that was more conserved in NBD2. These observations suggest that NBD1 may be the main ATP binding domain. Novel conservation pattern in NBD2 suggests that some functions may be unique to NBD2. Presence of the motifs required for ATP binding suggests that NBD2 is also capable of binding ATP. The NBD2 may either be required for the hydrolysis of ATP bound to NBD1, or acts as a backup domain for ATP binding. These predictions were supported by the ligand binding analysis (Table 1). *In silico* testing with a complete ABCB1 protein, ATP, ADP, and Mg showed preferential binding to NBD1. In the absence of NBD1, however, all three ligands showed binding to NBD2 as well (data not shown).

## Concluding remarks

The proposed sequence comparison criterion is important for the identification of true orthologs of a gene particularly belonging to a gene family. In maize, sorghum and Arabidopsis, the mutation in *ABCB1* gene resulted in altered plant architecture with reduced height due to the decreased auxin transport (Noh et al., 2001; Multani et al., 2003). The present comprehensive study provides the basic knowledge about the existence, structure and putative function of ABCB1 in crop plants, however, extensive characterization of these putative candidates of ABCB1 is required to establish their biological function in crop plants. Due to the similar structure and putative function of ABCB1 among studied crops, this gene can be used as a potential candidate to develop auxin based dwarf phenotypes in cereals.

## Author contributions

Contributions to the conception or design of the work; or the acquisition, analysis, or interpretation of data for the work—Amandeep K. Dhaliwal, Amita Mohan, Kulvinder S. Gill. Drafting the work or revising it critically for important intellectual content—Amandeep K. Dhaliwal, Amita Mohan, Kulvinder S. Gill. Final approval of the version to be published—Amandeep K. Dhaliwal, Amita Mohan, Kulvinder S. Gill. Agreement to be accountable for all aspects of the work in ensuring that questions related to the accuracy or integrity of any part of the work are appropriately investigated and resolved—Amandeep K. Dhaliwal, Amita Mohan, Kulvinder S. Gill.

### Conflict of interest statement

The authors declare that the research was conducted in the absence of any commercial or financial relationships that could be construed as a potential conflict of interest.
