# Supplementary material for: Comparative analysis of ABCB1 reveals novel structural and functional conservation between monocots and dicots
Source: Front Plant Sci. 2014 Nov 26;5:657. doi: 10.3389/fpls.2014.00657 (PMC4245006; doi:10.3389/fpls.2014.00657)
Supplement: Supplementary file 2 [file Presentation2.PDF]

A)

```
ZmABCB1  MQEQAHEAALVNARRSSARPSSARNSVSSPIM--TRNSSYGRSPYSRRLSDFSTSD-FTLSIHDPHH---HHRTMADKQLAFRAGASSFLRLARMNSPEWAYA
SbABCB1  MQEQAHEAAFVNARRSSARPSSARNSVSSPIM--TRNSSYGRSPYSRRLSDFSTSD-FTLSIHDPHH---HHRTMADKQLAFRAGASSFLRLARMNSPEWAYA
HvABCB1  MQEQAHEAALVSARRSSARPSSARNSVSSPIM--MRNSSYGRSPYSRRLSDFSTADFSLSVIHDP-AAHRMGM--GMEKLAFRAQASSFWRLAKMNSPEWGYA
TaABCB1  MQEQAHEAALVSARRSSARPSSARNSVSSPIM--MRNSSYGRSPYSRRLSDFSTADFSLSVIHDP-AAHRMGMGMGMEKLAFRAQASSFWRLAKMNSPEWGYA
OsABCB1  MQEQAHEAALVAARRSSARPSSARNSVSSPII--TRNSSYGRSPYSRRLSDADFITGLGLGVD---------SKQQQQQHYFRVQASSFWRLAKMNSPEWGYA
BdABCB1  MQEQAHDAA---ARRSSARPSSARNSVSSPIAMMQRNSSYGRSPYSRRLSDFSASDFGLSSSVVVMQQHDVHGGGMMKKLAFRAQASSFWRLAKMNSPELGYA
AtABCB1  MQEAAHETAMSNARKSSARPSSARNSVSSPIM--TRNSSYGRSPYSRRLSDFSTSDFSLSIDASS------YPNYRNEKLAFKDQANSFWRLAKMNSPEWKYA
GmABCB1  MQEMAHETAMNNARKSSARPSSARNSVSSPII--ARNSSYGRSPYSRRLSDFSTSDFSLSLDAS-------HPSYRLEKLAFKEQASSFWRLAKMNSPEWLYA
ZmABCB19 FQETARNRA--CPSTRKSRSSRLSNSLSTRSLSL-RSGSLRNLSYS-------YSTGADGRI-------E-MVSNADNDRKYPAPRGYFFKLLKLNAPEWPYT
SbABCB19 FQETARNRA--CPSTRKSRSSRLSNSLSTRSLSL-RSGSLRNLSYS-------YSTGADGRI-------E-MVSNADNDRKYPAPRGYFFKLLKLNAPEWPYT
HvABCB19 FQEMAKNRDFRGASTRKNRSSRLSNSLSTRSLSL-RSGSLRNLSYS-------YSTGADGRI-------E-MVSNADNDRKYPAPKGYFFKLLKLNAPEWPYT
TaABCB19 FQEMARNRDFRGASTRKNRSSRLSNSLSTRSLSL-RSGSLRNLSYS-------YSTGADGRI-------E-MVSNADNDRKYPAPKGYFFKLLKLNAPEWPYT
OsABCB19 FQEMARNRDFRGPSTRKSRSSRLSNSLSTRSLSL-RSGSLRNLSYS-------YSTGADGRI-------E-MVSNADNDRKYPAPKGYFFKLLKLNAPEWPYT
BdABCB19 FQEMARNRDFRGSSTRKNRSSRLSNSLSTRSLSL-RSGSLRNLSYS-------YSTGADGRI-------E-MVSNADNDRKYPAPKGYFFKLLKLNAPEWPYT
AtABCB19 FQEMVGTRDFSNPSTRRTRSTRLSHSLSTKSLSL-RSGSLRNLSYS-------YSTGADGRI-------E-MISNAETDRKTRAPENYFYRLLKLNSPEWPYS
GmABCB19 FQEMVGNRDFSNPSTRRTRSSRLSHSLSTKSLSL-RSGSLRNLSYQ-------YSTGADGRI-------E-MISNAETDKKNPAPDGYFFRLLKMNAPEWPYS
```

B)

```
AtABCB1  MQEAAHETAMSN-----ARKSSARPSSARNSVSSPIMTRNSSYGRSPYSRRLSDF---STSDFSLSIDASSYPNYRNEKLAFKDQANSFWRLAKMNSPEWKYA
AtABCB19 FQEMVGTRDFSN-----PSTRRTRSTRLSHSLSTKSLSLRSGSLRN-LSYSYSTG---ADGRIEMISNAETDRKTR-------APENYFYRLLKLNSPEWPYS
AtABCB14 CQDTEPQENLRS-----VMYESCRSQAGSYSSRRVFSSRRTSSFRE--DQEKTEK---DSKGEDLISSSSS----------------MIWELIKLNAPEWLYA
AtABCB15 LQQIEKQDINVS-----VKIGPISDPSKDIRNSSRVSTLSRSSSANSVTGPSTIK---NLSEDNKPQLPS------------------FKRLLAMNLPEWKQA
AtABCB4  LQEEKKSDENAAEEQKMSSIESFKQSSLRKSSLGRSLSKGGSSRGNSSRHSFNMFGFPAGIDGNVVQDQEEDDTTQPKTE---PKKVSIFRIAALNKPEIPVL
```

Fig. S2 – Alignment of linker regions of A) ABCB1 and ABCB19 proteins from maize (Zm), sorghum (Sb), barley (Hv), wheat (Ta), rice (Os), Brachypodium (Bd), Arabidopsis (At), and soybean (Gm); B) ABCB1, ABCB19, ABCB14, ABCB15, and ABCB4 from Arabidopsis.
