# Supplementary material for: Comparative analysis of ABCB1 reveals novel structural and functional conservation between monocots and dicots
Source: Front Plant Sci. 2014 Nov 26;5:657. doi: 10.3389/fpls.2014.00657 (PMC4245006; doi:10.3389/fpls.2014.00657)
Supplement: Supplementary file 3 [file Table1.DOC]

| **Species** | **Intron** | | | | | | | | |
| --- | --- | --- | --- | --- | --- | --- | --- | --- | --- |
|  | **1** | **2** | **3** | **4** | **5** | **6** | **7** | **8** | **9** |
| Maize | 605-748 | 1386-1478 | 1786-2399 | 2630-4292 |  |  |  |  |  |
|  | (144) | (93) | (613) | (1663) |  |  |  |  |  |
| Sorghum | 617-781 | 1419-1528 | 1855-2700 | 2931-4401 |  |  |  |  |  |
|  | (165) | (110) | (846) | (1471) |  |  |  |  |  |
| Barley | 1212-1314 | 1632-1730 | 1955-3005 |  |  |  |  |  |  |
|  | (103) | (99) | (1051) |  |  |  |  |  |  |
| Wheat | 1193-1318 | 1627-1723 | 1950-2957 |  |  |  |  |  |  |
|  | (126) | (97) | (1008) |  |  |  |  |  |  |
| Rice | 1155-1248 | 1784-2935 |  |  |  |  |  |  |  |
|  | (94) | (1152) |  |  |  |  |  |  |  |
| Brachypodium | 1212-1292 | 1834-2920 |  |  |  |  |  |  |  |
|  | (81) | (1087) |  |  |  |  |  |  |  |
| Arabidopsis | 265-333 | 389-473 | 650-722 | 924-1050 | 1311-1409 | 1727-1813 | 2038-2365 | 2740-2822 | 3541-3617 |
|  | (69) | (85) | (73) | (127) | (99) | (87) | (328) | (83) | (77) |
| Soybean | 415-536 | 592-687 | 864-967 | 1169-1263 | 1524-1629 | 1947-2068 | 2293-3879 | 4254-4395 | 5111-5194 |
|  | (122) | (96) | (104) | (95) | (106) | (122) | (1587) | (142) | (84) |

**Supplementary Table 1.** The position and size (in parenthesis) of introns in different species. Position and size are given in base pairs. The intron position is from marked from translation start site.
