# Supplementary material for: Comparative analysis of ABCB1 reveals novel structural and functional conservation between monocots and dicots
Source: Front Plant Sci. 2014 Nov 26;5:657. doi: 10.3389/fpls.2014.00657 (PMC4245006; doi:10.3389/fpls.2014.00657)
Supplement: Supplementary file 4 [file Table2.DOC]

| **Species** | **% Nucleotide identity with Maize** | | | | | |
| --- | --- | --- | --- | --- | --- | --- |
|  | **Gene** | **Exon1** | **Exon2** | **Exon3** | **Exon4** | **Exon5** |
| Sorghum | 90.0 | 84.0 | 94.5 | 90.8 | 91.3 | 95.6 |
| Barley | 80.0 | 73.6 | 86.3 | 81.4 | 84.1 | 85.2 |
| Wheat | 80.0 | 73.6 | 87.1 | 81.4 | 85.0 | 85.1 |
| Rice | 78.0 | 67.5 | 87.1 | 82.0 | 83.9 | 83.2 |
| Brachypodium | 78.0 | 68.4 | 84.2 | 78.7 | 80.9 | 80.0 |
| Arabidopsis | 62.0 | 63.6 | 65.3 | 65.6 | 67.8 | 67.5 |
| Soybean | 56.0 | 61.2 | 69.4 | 67.8 | 67.2 | 64.6 |

**Supplementary Table 2.** Nucleotide percent identity for gene and their respective exons in different species with respect to maize. Percent identity with respect to maize exons was calculated for corresponding exonic regions in different species.
