# Supplementary material for: Comparative analysis of ABCB1 reveals novel structural and functional conservation between monocots and dicots
Source: Front Plant Sci. 2014 Nov 26;5:657. doi: 10.3389/fpls.2014.00657 (PMC4245006; doi:10.3389/fpls.2014.00657)
Supplement: Supplementary file 5 [file Table3.DOC]

| **Species** | **% Amino acid identity** | | |
| --- | --- | --- | --- |
|  | **With Maize** | **TMD1-2** | **NBD1-2** |
| Maize |  | 25.8 | 55.1 |
| Sorghum | 91.0 | 21.2 | 56.6 |
| Barley | 83.0 | 25.4 | 55.4 |
| Wheat | 86.0 | 25.1 | 55.4 |
| Rice | 85.0 | 26.2 | 59.3 |
| Brachypodium | 79.0 | 28.3 | 58.5 |
| Arabidopsis | 75.0 | 23.4 | 48.1 |
| Soybean | 76.0 | 24.1 | 60.4 |

**Supplementary Table 3.** Amino acid percent identity of predicted proteins in different species with respect to maize along with sequence comparison between TMD1 and 2 and NBD 1 and 2 within each species.
