# Supplementary material for: Comparative analysis of ABCB1 reveals novel structural and functional conservation between monocots and dicots
Source: Front Plant Sci. 2014 Nov 26;5:657. doi: 10.3389/fpls.2014.00657 (PMC4245006; doi:10.3389/fpls.2014.00657)
Supplement: Supplementary file 6 [file Table4.DOC]

| **Species** | **ZmABCB1** | | |
| --- | --- | --- | --- |
|  | **Coverage (ABCB1)** | **Coverage (ABCB19)** | **Identity (ABCB19)** |
| Sorghum | 99 | 89 | 52 |
| Barley | 97 | 89 | 52 |
| Wheat  Rice | 97  88 | 89  89 | 52  52 |
| Brachypodium | 97 | 89 | 52 |
| Arabidopsis | 91 | 91 | 51 |
| Soybean | 91 | 91 | 51 |

**Supplementary Table 4.** Amino acid percent coverage of predicted ABCB1 and coverage and identity of predicted ABCB19 protein in different species with respect to ZmABCB1.
