# Supplementary material for: Comparative analysis of ABCB1 reveals novel structural and functional conservation between monocots and dicots
Source: Front Plant Sci. 2014 Nov 26;5:657. doi: 10.3389/fpls.2014.00657 (PMC4245006; doi:10.3389/fpls.2014.00657)
Supplement: Supplementary file 7 [file Table5.DOC]

| AtABCB1 | GmABCB1 | ZmABCB1 | SbABCB1 | HvABCB1 | TaABCB1 | OsABCB1 | BdABCB1 |
| --- | --- | --- | --- | --- | --- | --- | --- |
| L (58) | L (108) | L (153) | L (157) | L (143) | L (144) | L (124) | L (142) |
| F (65) | F (115) | F (160) | F (164) | F (150) | F (151) | F (131) | F (149) |
| V (198) | V (248) | V (293) | V (297) | V (283) | V (284) | V (264) | V (283) |
| Y (279) | Y (329) | Y (374) | Y (378) | Y (364) | Y (365) | Y (345) | Y (364) |
| F (280) | F (330) | F (375) | F (379) | F (365) | F (366) | F (346) | F (365) |
| V (282) | V (332) | V (377) | V (381) | V (367) | V (368) | V (348) | V (367) |
| F (283) | F (333) | F (378) | F (382) | F (368) | F (369) | F (349) | F (368) |
| Y (286) | Y (336) | Y (381) | Y (385) | Y (371) | Y (372) | Y (352) | Y (371) |
| A (287) | A (337) | G (382) | G (386) | A (372) | A (373) | A (353) | A (372) |
| G (305) | G (355) | G (400) | G (404) | G (390) | G (391) | G (371) | G (390) |
| M (311) | M (361) | M (406) | M (410) | M (396) | M (397) | M (377) | M (396) |
| F (312) | F (362) | F (407) | F (411) | F (397) | F (398) | F (378) | F (397) |
| M (315) | M (365) | M (410) | M (414) | M (400) | M (401) | M (381) | M (400) |
| I (316) | I (366) | I (411) | I (415) | I (401) | I (402) | I (382) | I (401) |
| G (318) | G (368) | G (413) | G (417) | G (403) | G (404) | G (384) | G (403) |
| L (319) | L (369) | L (414) | L (418) | L (404) | L (405) | L (385) | L (404) |
| Q (323) | Q (373) | Q (417) | Q (422) | Q (408) | Q (409) | Q (389) | Q (408) |
| C (710) | C (759) | C (807) | C (816) | C (798) | C (798) | C (770) | C (800) |
| G (711) | G (760) | G (808) | G (817) | G (799) | G (799) | G (771) | G (801) |
| S (712) | S (761) | S (809) | S (818) | S (800) | S (800) | S (772) | S (802) |
| L (713) | L (762) | F (810) | F (819) | F (801) | F (801) | F (773) | M (803) |
| S (714) | S (763) | S (811) | S (820) | S (802) | S (802) | S (774) | S (804) |
| F (716) | F (765) | I (813) | I (822) | I (804) | I (804) | I (776) | V (806) |
| F (717) | F (766) | F (814) | F (823) | F (805) | F (805) | F (777) | F (807) |
| V (720) | V (769) | I (817) | I (826) | I (808) | I (808) | V (780) | I (810) |
| S (752) | S (801) | S (849) | S (858) | S (840) | S (840) | S (812) | S (842) |
| A (753) | T (802) | A (850) | A (859) | A (841) | A (841) | A (813) | A (843) |
| V (756) | L (805) | L (853) | L (862) | L (844) | L (844) | L (816) | L (846) |
| A (829) | A (878) | A (926) | A (935) | A (917) | A (917) | A (889) | A (919) |
| V (852) | V (901) | V (949) | V (958) | V (940) | V (940) | V (912) | V (942) |
| F (853) | F (902) | F (950) | F (959) | F (941) | F (941) | F (913) | F (943) |
| V (856) | V (905) | V (953) | V (962) | V (944) | V (944) | V (916) | V (946) |
| T (860) | T (909) | T (957) | T (966) | T (948) | T (948) | T (920) | T (950) |
| Y (930) | Y (979) | Y (1027) | Y (1036) | Y (1018) | Y (1018) | Y (990) | Y (1020) |
| A (933) | A (982) | A (1030) | A (1039) | A (1021) | A (1021) | A (993) | A (1023) |
| Q (934) | Q (983) | Q (1031) | Q (1040) | Q (1022) | Q (1022) | Q (994) | Q (1024) |
| L (937) | L (986) | L (1034) | L (1043) | L (1025) | L (1025) | L (997) | L (1027) |
| Y (938) | Y (987) | Y (1035) | Y (1044) | Y (1026) | Y (1026) | Y (998) | Y (1028) |
| S (940) | S (989) | S (1037) | S (1046) | S (1028) | S (1028) | S (1000) | S (1030) |
| Y (941) | Y (990) | Y (1038) | Y (1047) | Y (1029) | Y (1029) | Y (1001) | Y (1031) |
| L (945) | L (994) | L (1042) | L (1051) | L (1033) | L (1033) | L (1005) | L (1035) |
| I (963) | I (1012) | I (1060) | I (1069) | I (1051) | I (1051) | I (1023) | I (1053) |
| F (966) | F (1015) | F (1063) | F (1072) | F (1054) | F (1054) | F (1026) | F (1056) |
| L (969) | L (1018) | L (1066) | L (1075) | L (1057) | L (1057) | L (1029) | L (1059) |
| M (970) | M (1019) | M (1067) | M (1076) | M (1058) | M (1058) | M (1030) | M (1060) |
| V (971) | V (1020) | V (1068) | V (1077) | V (1059) | V (1059) | V (1031) | V (1061) |
| L (972) | L (1021) | L (1069) | L (1078) | L (1060) | L (1060) | L (1032) | L (1062) |
| M (973) | M (1022) | M (1070) | M (1079) | M (1061) | M (1061) | M (1033) | M (1063) |
| V (974) | V (1023) | V (1071) | V (1080) | V (1062) | V (1062) | V (1034) | V (1064) |
| A (976) | A (1025) | A (1073) | A (1082) | A (1064) | A (1064) | A (1036) | A (1066) |
| A (977) | A (1026) | A (1074) | A (1083) | A (1065) | A (1065) | A (1037) | A (1067) |
| L (980) | L (1029) | L (1077) | L (1086) | L (1068) | L (1068) | L (1040) | L (1070) |

Supplementary Table 5 – Amino acids predicted in auxin binding. The residues are predicted based on their conservation with AtABCB1 (Bailly et al., 2011) and their positions are given in parenthesis.
